# HIPK2 sustains apoptotic response by phosphorylating Che-1/AATF and promoting its degradation

**DOI:** 10.1038/cddis.2014.381

**Published:** 2014-09-11

**Authors:** F De Nicola, V Catena, C Rinaldo, T Bruno, S Iezzi, C Sorino, A Desantis, S Camerini, M Crescenzi, A Floridi, C Passananti, S Soddu, M Fanciulli

**Affiliations:** 1 Epigenetics Laboratory, Regina Elena National Cancer Institute, Rome, Italy; 2 Department of Biothecnological and Applied Clinical Science, University of L'Aquila, Aquila, Italy; 3 Institute of Molecular Biology and Pathology, National Research Council (CNR), c/o Sapienza University of Rome, Rome, Italy; 4 Experimental Oncology Laboratory, Regina Elena National Cancer Institute, Rome, Italy; 5 Department of Cell Biology and Neurosciences, Higher Institute of Health (ISS), Rome, Italy

## Abstract

Che-1/AATF is an RNA polymerase II-binding protein that is involved in the regulation of gene transcription, which undergoes stabilization and accumulation in response to DNA damage. We have previously demonstrated that following apoptotic induction, Che-1 protein levels are downregulated through its interaction with the E3 ligase HDM2, which leads to Che-1 degradation by ubiquitylation. This interaction is mediated by Pin1, which determines a phosphorylation-dependent conformational change. Here we demonstrate that HIPK2, a proapoptotic kinase, is involved in Che-1 degradation. HIPK2 interacts with Che-1 and, upon genotoxic stress, phosphorylates it at specific residues. This event strongly increases HDM2/Che-1 interaction and degradation of Che-1 protein via ubiquitin-dependent proteasomal system. In agreement with these findings, we found that HIPK2 depletion strongly decreases Che-1 ubiquitylation and degradation. Notably, Che-1 overexpression strongly counteracts HIPK2-induced apoptosis. Our results establish Che-1 as a new HIPK2 target and confirm its important role in the cellular response to DNA damage.

Che-1/AATF (Che-1) is a highly conserved RNA polymerase II (Pol II) binding protein that regulates gene transcription and cell proliferation.^1, 2, 3, 4, 5^ Che-1 interacts with Rb and interferes with the Rb-mediated recruitment of histone deacetylase I on the promoters of E2F1-responsive genes, thus inhibiting the Rb growth-suppressing functions.^1, 6^ In agreement, the mouse ortholog of Che-1, Traube, is essential for proliferation in early embryogenesis.^2^ Most of the Che-1 functions are regulated by posttranslational modifications that determine the interaction specificity and protein stability in response to DNA damage.^7, 8, 9^ Moreover, a recent study demonstrated that, upon genotoxic stress, Che-1 is phosphorylated by the checkpoint kinase MK2, and this event results in nuclear translocation where Che-1 binds to the PUMA, BAX and BAK promoter regions to repress expression of these proapoptotic genes.^10^ In addition to the pro-proliferative function, Che-1 exhibits strong anti-apoptotic activity^4, 11, 12^ and is downregulated during apoptosis through its interaction with HDM2/MDM2 human double minute 2, E3 ubiquitin protein ligase (HDM2)^9^ and NRAGE.^13^ In particular, in response to apoptotic induction, Che-1 is phosphorylated at threonine 144 (T144), and this modification is required for Pin1-mediated modifications and HDM2 ubiquitylation.^9^ Consistent with these findings, a Che-1 mutant lacking T144, and therefore unable to bind Pin1, results in less degradation and counteracts apoptosis more efficiently than wild-type protein.^9^

Homeodomain-interacting protein kinase 2 (HIPK2) is a Tyr-activated Ser/Thr kinase.^14, 15, 16^ It is a multifunctional kinase that by virtue of protein/protein interaction with a still growing list of targets and phosphorylation of specific S/T residues regulates gene transcription and the response to DNA damage.^17, 18, 19, 20^ Different genotoxic stimuli, such as ultraviolet (UV),^21, 22^ or anti-neoplastic treatments, such as Adriamycin (ADR)^23^ and cisplatin,^24^ activate HIPK2, which then specifically phosphorylates human p53 at Ser46,^21, 22^ a posttranscriptional modification that leads to apoptosis.^25^ Indeed, HIPK2-induced p53 Ser46 phosphorylation promotes the activation of proapoptotic factors, such as p53AIP1, BAX, PIG3, Noxa, Puma, KILLER/DR5 and PTEN,^21, 24, 26^ as well as the repression of the anti-apoptotic factor galectin-3,^27^ rather than the transcription of the gene responsible for growth arrest.^25, 28^ Furthermore, p53 Ser46 phosphorylation by HIPK2 changes the interaction between HDM2 and p53, prevents the HDM2-mediated cytoplasmic shuttling and ubiquitylation of p53 in response to genotoxic agents and recovers the p53-apoptotic functions.^24^ Moreover, HIPK2 can induce apoptosis in a p53-independent manner by promoting a phosphorylation-dependent proteasomal degradation of the anti-apoptotic transcriptional co-repressor C-terminal binding protein (CtBP)^29^ and ΔNp63*α*, a prosurvival ΔN isoform of the p53 family member that is directly phosphorylated by HIPK2, and this kinase activity promotes proteasomal degradation of ΔNp63*α* in response to anticancer therapy.^30^ In agreement with these findings, in less severe and presumably reparable DNA-damage conditions, HIPK2 is ubiquitylated by HDM2 in a p53-dependent manner and targeted to proteasomal degradation,^23^ and two different HDM2 antagonists, Nutlin-3 and reactivating p53 and inducing tumor apoptosis, regulate HIPK2 inducing mitotic arrest or apoptosis.^31^

In this study, we identify Che-1 as a novel target of HIPK2 activity. Indeed, we show that HIPK2 and Che-1 interact and that this binding increases in response to DNA damage. Notably, we found that HIPK2 phosphorylates a specific residue of Che-1, which is required for its interaction with Pin1 and for its degradation through the proteasome pathway. Consistent with these findings, we provide evidence that HIPK2 expression strongly affects Che-1 protein levels and that Che-1 is able to counteract apoptotic activity of HIPK2.

## Results

### Che-1 interacts with HIPK2 in response to apoptosis

**Figure 1:**
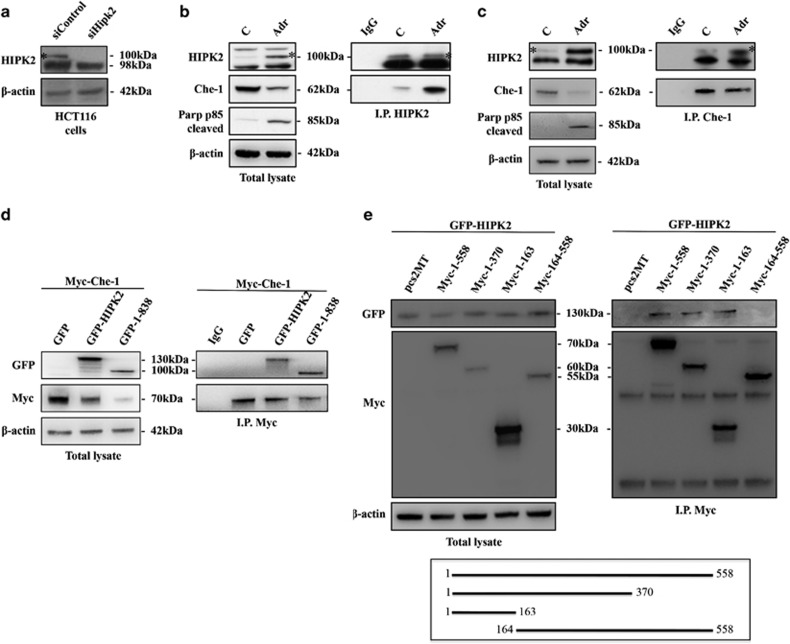
*Che-1 interacts with HIPK2 in response to apoptosis.* (**a**) HCT116 cells were transiently transfected with siRNA (siControl) or siRNA HIPK2 (siHIPK2), and 24 h later the total cell extracts (TCEs) were analyzed by western blotting (WB) with the indicated antibodies (Abs). (**b** and **c**) HCT116 cells were treated for 24 h with 2 *μ*M ADR. TCEs were immunoprecipitated with anti-HIPK2 (**b**) or anti-Che-1 (**c**) antibodies and analyzed by WB using the indicated antibodies. (**d**) HCT116 cells were transiently transfected with expression vectors Myc-Che-1, GFP-HIPK2 and its deletion mutant. TCEs were immunoprecipitated with anti-Myc antibody and analyzed by WB using the indicated Abs. (**e**) HCT116 cells were transfected with expression vectors GFP-HIPK2, Myc-Che-1 and its deletion mutants. TCEs were immunoprecipitated and analyzed as in **d**. The bottom panel shows a schematic representation of full-length Che-1 protein and its deletion mutants

As described above, Che-1 phosphorylation at T144 seems to have an important role in the apoptosis process, and as this residue is a canonical motif for HIPK2 phosphorylation we wondered whether Che-1 could be a new HIPK2 target during apoptosis activation. For this reason, we started to analyze the role of HIPK2 in this modification by testing whether the endogenous HIPK2 and Che-1 proteins physically interact. As the anti-HIPK2 antibody shows several unspecific background bands,^32^ we performed HIPK2 depletion in HCT116 cells by siRNA to identify the specific signal (Figure 1a). As shown in Figure 1b, Che-1 co-precipitates with HIPK2 in control cells and, notably, the amount of Che-1 co-precipitating with HIPK2 strongly increases in response to an apoptotic dose of ADR. As expected, an increase of HIPK2 expression and a concomitant decrease of Che-1 protein levels occur in HCT116 cells after ADR treatment^9, 23^ (Figure 1b). In agreement, a coimmunoprecipitation performed with anti-Che-1 antibody confirmed Che-1/HIPK2 interaction (Figure 1c). Furthermore, we observed that exogenous Myc-tagged Che-1 co-precipitates with exogenous GFP-tagged HIPK2 and, consistent with the results obtained with endogenous protein, exogenous Che-1 showed a greater affinity for the caspases-cleaved, active form of HIPK2 (1–838)^33^ compared with wt (Figure 1d) Finally, an analysis performed with several Che-1 mutants revealed that the region 1–163 of this protein is required for its interaction with HIPK2 (Figure 1e). Together, these findings indicate that the two proteins belong to the same complex and that apoptotic induction increases the presence of Che-1 in this complex.

### HIPK2 phosphorylates Che-1

**Figure 2:**
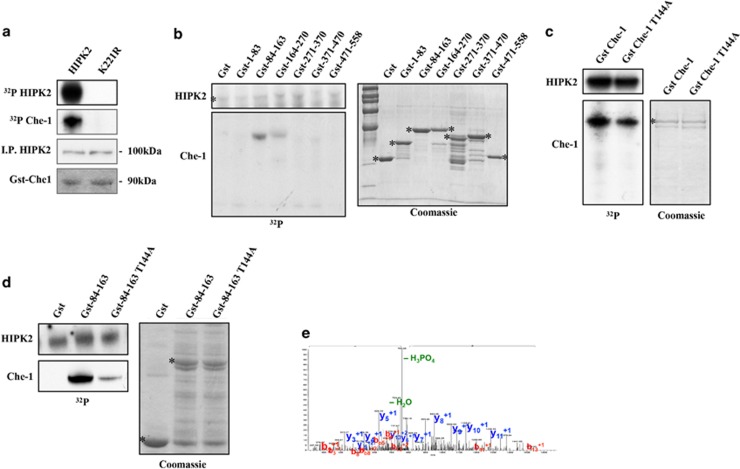
*HIPK2 phosphorylates Che-1.* (**a**) HIPK2 wt or its kinase-defective form K221R were immunoprecipitated from transfected HEK293 cells and used to phosphorylate purified Gst-Che-1 protein in *in vitro* reactions in the presence of [*γ*^32^P]-ATP. The kinase reaction products were resolved by Sodium dodecyl sulphate-polyacrylamide gel electrophoresis and analyzed by autoradiography (upper panel). WB analysis was performed after gel rehydration with the indicated Abs. (**b**, **c** and **d**) Gst-Che-1 or its mutant proteins were used as substrate for HIPK2 kinase assays. The left panels depict HIPK2 and Che-1 ^32^P autoradiographs, whereas the right panels show Coomassie blue stain of purified Gst-Che-1 proteins. (**e**) The panel shows the MS/MS spectrum of the phosphopeptide ***p*****T**PGFSVQSISDFEK (precursor ion (MH_2_)^2+^ 811.37) (***p*****T** is for phosphorylated threonine). Detected peaks, corresponding to the ions of the *b* and *y* series, are labeled and indicated in red and blue, respectively. The ion at m/z 762.4 is due to 49 Da neutral loss, related to a phosphoric acid molecule, and it demonstrates the presence of a phosphorylated peptide. The presence of the complete *y*-series without any mass increase and the occurrence of the phosphorylated b_2_ ion confirm the localization of the phosphorylation on the threonine 144

As previously demonstrated, HIPK2 regulates several of its targets through the kinase activity.^18^ We then examined whether Che-1 is a target of HIPK2 kinase activity by *in vitro* kinase assays. Recombinant glutathione S-transferase (Gst)-Che-1 was used as a substrate for HIPK2 immunoprecipitated from HEK293 cells. The presence of a phosphorylation signal on Gst-Che-1 indicates that Che-1 can be phosphorylated by HIPK2 *in vitro*, whereas the absence of Che-1 phosphorylation, as well as HIPK2 autophosphorylation by the kinase-defective form of HIPK2 (K221R), demonstrates the specificity of these reactions (Figure 2a). To identify the Che-1 residue/s target of HIPK2, Gst-fusion polypeptides covering the whole Che-1 protein were used as substrates in *in vitro* kinase assays with HIPK2. As shown in Figure 2b, HIPK2 was able to phosphorylate the peptide Gst-84-163, and to a lesser extent the peptide Gst-164-270, suggesting that HIPK2 targets more than one residue on Che-1. Interestingly, the peptide Gst-84-163 contains the residue T144, a consensus site for HIPK2 phosphorylation and a critical residue for Che-1 degradation mediated by HDM2 and regulated by Pin1.^9^ To evaluate whether T144 is one of the target sites of HIPK2 phosphorylation, we used Gst-Che-1 and its derivative, in which T144 is substituted with an alanine (Gst-Che-1^T144A^), as substrates in *in vitro* kinase assays with HIPK2. We observed a significant reduction of Gst-Che-1^T144A^ phosphorylation by HIPK2 compared with that of Gst-Che-1 wt (Figure 2c), and these results were confirmed by a kinase assay performed with Gst-84-163 wt and Gst-84-163^T144A^ as substrates (Figure 2d). Notably, these results were confirmed by a Mass-spectroscopy analysis that showed Che-1 T144 as a target of HIPK2 (Figure 2e). Altogether, these results demonstrate that HIPK2 phosphorylates T144 of Che-1.

### HIPK2 induces Che-1 degradation

**Figure 3:**
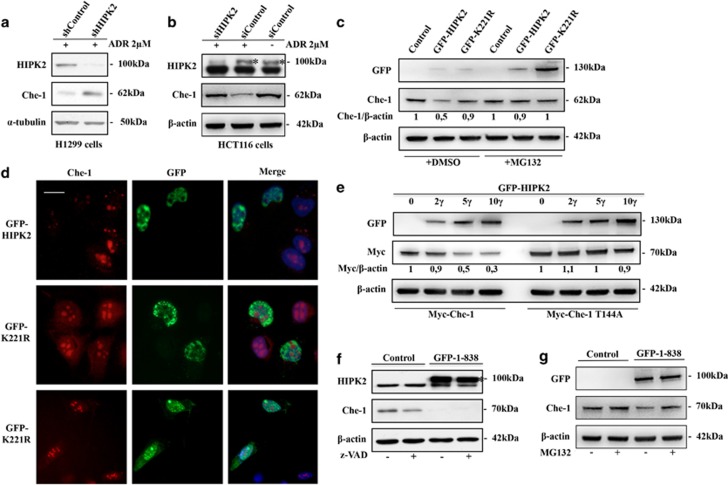
*HIPK2 induces Che-1 degradation.* (**a** and **b**) H1299 (A) or HCT116 (**b**) cells were transiently transfected with shControl or shHIPK2 (A), or siRNA GFP (siControl) or siRNA HIPK2 (**b**), and treated with 2 *μ*M ADR. TCEs were analyzed by WB with the indicated Abs. (**c**) HCT116 cells were transiently transfected with GFP-HIPK2 or GFP-K221R expression vectors and treated or not treated with 10 *μ*M MG132 for 16 h. TCEs were analyzed by WB with the indicated Abs. (**d**) U2OS cells were transiently transfected as in **c** and analyzed by immunofluorescence with anti-Che-1 antibody. (**e**) HCT116 cells were transiently transfected with Myc-Che-1 or Myc-Che-1^T144A^ and increasing amounts of GFP-HIPK2. TCEs were analyzed by WB with the indicated Abs. (**f** and **g**) HCT116 cells were transiently transfected with HIPK2 (1–838) expression vector and treated or not treated with 50 *μ*M z-VAD for 12 h (F) or 10 *μ*M MG132 for 16 h (G). TCEs were analyzed by WB with the indicated Abs

As described, in response to apoptotic stimuli, Che-1 binds Pin1, which, in turn, mediates conformational changes of Che-1.^9^ This event is required for Che-1/HDM2 interaction with consequent Che-1 degradation, and it depends on the phosphorylation at residue T144. Indeed, substitution of T144 reduces the ability of Che-1 to bind both Pin1 and HDM2, with an increase of its half-life.^9^ On the basis of these observations, we speculated that HIPK2 might be involved in Che-1 stabilization. To test this hypothesis, we performed a western blot analysis of the total cell extracts (TCEs) obtained from H1299 and HCT116 cells that were transiently transfected with HIPK2 shRNA or siRNA and treated with ADR. As is shown in Figures 3a and b, HIPK2 depletion strongly increased Che-1 protein levels. In agreement with these findings, HIPK2 overexpression was able to reduce Che-1 levels, whereas this effect was not observed in cells transfected with K221R mutant (Figure 3c). Notably, MG132 treatment counteracted HIPK2 activity on Che-1 (Figure 3c), thus reinforcing the notion that HIPK2 affects Che-1 stability. These results were confirmed by immunofluorescence assays that revealed the absence of Che-1 protein in U2OS cells overexpressing HIPK2 but not its K221R derivative (Figure 3d). To confirm the involvement of T144 in Che-1 degradation by HIPK2, we transiently transfected HCT116 cells with vectors carrying the Che-1 wt or Che-1^T144A^ mutant and increasing amounts of vector carrying HIPK2. As shown in Figure 3e, HIPK2 overexpression strongly reduced Myc-Che-1 levels, whereas it produced little effect on Che-1^T144A^ expression. Next, to evaluate whether Che-1 degradation is due to direct modification by HIPK2 and not rather the result of apoptotic induction, we treated HCT116 cells with a specific inhibitor of caspases (z-VAD) in the presence or absence of the C-terminal-deleted form of HIPK2 (1–838) that lacks the autoinhibitory domain and mimics the caspase-superactivated form of HIPK2.^33^ As shown in Figure 3f, a significant Che-1 degradation was observed in cells overexpressing superactivated HIPK2 (1–838) in the presence of z-VAD but not in the presence of MG132 (Figure 3g), confirming in such a way that HIPK2 is directly involved in Che-1 degradation.

### HIPK2 promotes Che-1 ubiquitylation

**Figure 4:**
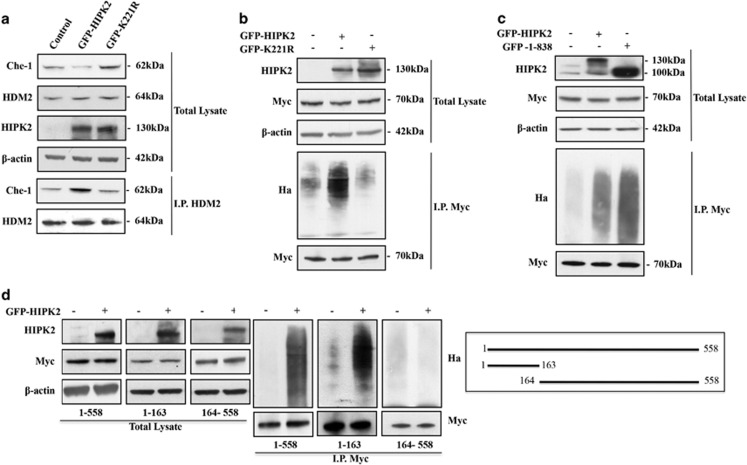
*HIPK2 promotes Che-1 ubiquitylation.* (**a**) HCT116 cells were transiently transfected with GFP-HIPK2 or GFP-K221R expression vectors. TCEs were immunoprecipitated with anti-MDM2 antibody and analyzed by WB with the indicated Abs. (**b**) HEK293 cells were co-transfected with Myc-Che-1, Ha-Ubiquitin and GFP-HIPK2 or GFP-K221R and treated with 10 *μ*M MG132 for 16 h. TCEs were immunoprecipitated with anti-Myc monoclonal antibody and analyzed by WB with the indicated Abs. (**c**) HEK293 cells transfected with Myc-Che-1, Ha-Ubiquitin and GFP-HIPK2 or GFP-HIPK2 (1–838) expression vectors and treated with MG132 as in B. TCEs were immunoprecipitated as in B and analyzed by WB with the indicated Abs. (**d**) HEK293 cells were transiently transfected with indicated Che-1 deletion mutants, Ha-Ubiquitin and GFP-HIPK2, and treated with MG132 as in B. TCEs were immunoprecipitated as in **b** and analyzed by WB with the indicated Abs. In the right panel, a schematic representation of full-length Che-1 protein and two of its deletion mutants is provided

As previously described, Che-1 half-life is tightly regulated by the proteasome pathway, and in response to apoptotic stimuli HDM2 protein negatively regulates Che-1 by promoting its ubiquitin-mediated degradation.^9^ On the basis of the above results, we evaluated whether HIPK2 could affect Che-1/HDM2 interaction by coimmunoprecipitation with anti-HDM2 antibody in HCT116 cells overexpressing HIPK2 or its K221R mutant and treated with ADR. As shown in Figure 4a, HCT116 cells overexpressing HIPK2 wt showed an increase of Che-1 bound to HDM2, whereas this effect was not observed in cells transfected with the K221R mutant. Consistent with these findings, HIPK2 overexpression produced a strong increase in the ubiquitylation of Che-1, which is not found in cells overexpressing the K221R mutant (Figure 4b), indicating that the kinase activity of HIPK2 is required for the interaction between Che-1 and HDM2 and Che-1 ubiquitylation. Notably, the superactive form of HIPK2 (1–838) was found to be more active in Che-1 ubiquitylation (Figure 4c), reinforcing the involvement of HIPK2 in Che-1 degradation in response to apoptotic induction. To further characterize the role of HIPK2 in Che-1 degradation, we transiently transfected HCT116 cells with HIPK2 and Che-1 in the wt form or two different deletion mutants (1–163 and 164–558). In agreement with previous results, we found that exogenous HIPK2 expression produces a strong ubiquitylation of Che-1 wt and 1–163 mutant, whereas the polypeptide 164–558 lacking T144 did not exhibit any ubiquitylation (Figure 4d). Taken together, these results support the hypothesis that upon induction of apoptosis HIPK2 phosphorylates Che-1, thereby increasing its affinity for HDM2, its ubiquitylation and its degradation.

### HIPK2 induces apoptosis by sustaining Che-1 degradation

**Figure 5:**
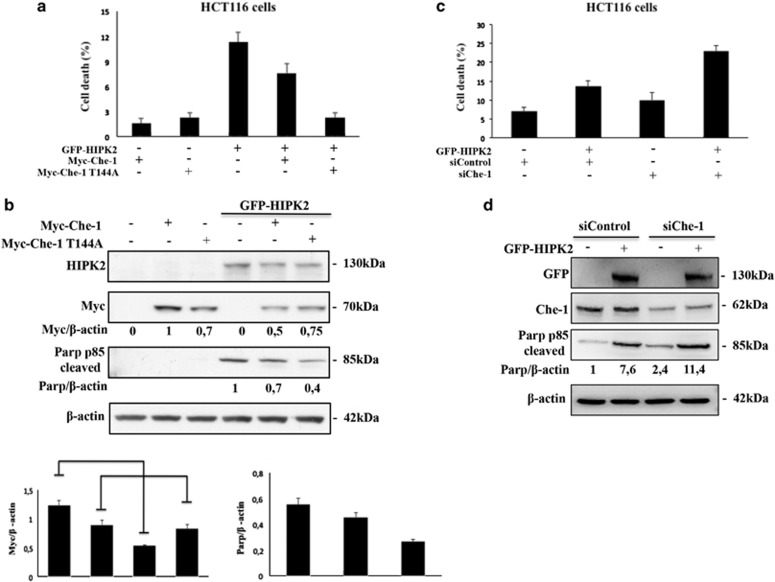
*HIPK2 induces apoptosis by sustaining Che-1 degradation.* (**a**) Cell death analysis of HCT116 cells transfected with Myc-Che-1 or Myc-Che-1^T144A^ in presence or absence of GFP-HIPK2 expression vectors. Twenty-four hours later, cell death was assayed by trypan blue staining, and percentages represent trypan blue-incorporating cells. Columns are average of three independent experiments, and error bars indicate standard deviation. (**b**) The top panel shows WB analysis of TCEs of **a** with indicated Abs. In the bottom panels is shown the schematic representation of Myc-Che-1 and Cleaved-PARP protein levels. Columns are average of five independent experiments, and error bars indicate standard deviation. (**c**) HCT116 cells were transiently transfected with siControl or siChe-1 in the presence or absence of GFP-HIPK2 expression vector. Twenty-four hours later, cell death was assayed as in **a**. (**d**) TCEs from **c** were analyzed by WB with the indicated Abs

The data described above prompted us to evaluate whether the proapoptotic activity of HIPK2 is also exerted through Che-1 phosphorylation and degradation. To this aim, we examined the effects of Che-1 wt or Che-1^T144A^ mutant overexpression on apoptosis induced by HIPK2. As shown in Figures 5a and b, the overexpression of Che-1 wt significantly counteracted the apoptotic activity of HIPK2, whereas the Che-1^T144A^ mutant exhibited a greater effect. Consistent with these results, Che-1^T144A^ mutant protein levels were not affected by HIPK2 overexpression. Conversely, Che-1 depletion amplified the apoptotic effect induced by HIPK2 overexpression (Figure 5c), and increased the levels of cleaved PARP (Figure 5d), confirming in such a way that Che-1 phosphorylation by HIPK2 and subsequent ubiquitylation has an important role in the induction of apoptosis.

## Discussion

Lethal doses of DNA-damaging agents trigger the apoptotic pathway by the enzymatic activation of caspases. During this event, the kinase HIPK2 is activated by a caspase-dependent cleavage of the C-terminal region that generates a highly active fragment that amplifies the apoptotic pathway. The kinase activity of HIPK2 contributes to the choice between cell cycle arrest and apoptosis, stabilizing proapoptotic factors such as p53^21, 22^ and degrading anti-apoptotic factors such as CtBP and ΔNp63*α*.^29, 30^

We have previously shown that, in response to genotoxic stresses, the half-life of the transcriptional regulator Che-1 is tightly regulated by the proteasome, and that specific phosphorylation protects this protein from degradation.^7^ Moreover, in response to apoptotic stimuli, Pin1 and HDM2 proteins negatively regulate Che-1 by promoting its ubiquitin-mediated degradation.^9^

In this study, we demonstrate that Che-1 is a direct target of HIPK2 and that their physical interaction is increased following the induction of apoptosis. We show that HIPK2 phosphorylates Che-1 at T144, an important residue involved in modifying the Che-1 structure and increasing its capacity to interact with HDM2.^9^ Consistent with these results, we also report that HDM2-Che-1 interaction is regulated by HIPK2. Indeed, HIPK2 overexpression strongly increases HDM2/Che-1 interaction, resulting in Che-1 ubiquitylation and degradation. Finally, we demonstrate that overexpression of Che-1^T144A^ mutant counteracts HIPK2-induced apoptosis more efficiently than Che-1 wt.

Recent studies have demonstrated that Che-1 exhibits a strong anti-apoptotic activity at least in part by regulating the expression of XIAP, an inhibitor of the enzymatic activity of caspases involved in apoptosis activation.^34^ AATF, the rat Che-1 orthologous gene, antagonizes Par-4 (prostate apoptosis response-4)-induced apoptosis, which is associated with neuronal degeneration observed in Alzheimer's disease.^12^

In agreement with these findings, Che-1 degradation is required for executing the apoptotic program.^35^ In particular, both HDM2 and NRAGE are able to downregulate Che-1 levels by targeting it for proteasome-dependent degradation in response to apoptotic stimuli.

Most aspects of HIPK2-induced apoptosis rely on its ability to reprogram the transcriptional response,^17^ regulating the levels of transcriptional activators or repressors in response to DNA damage, such as CtBP,^29^ or inducing the proapoptotic effects of p53.^21^ Therefore, it is possible to consider its effects on a coactivator factor such as Che-1 from this functional point of view, underscoring its role as a regulator of several different molecules. Furthermore, it has been recently shown that following DNA damage Che-1 is phosphorylated by p38MAPK/MK2. This phosphorylation results in the disruption of cytoplasmic MRLC/Che-1 complexes, allowing Che-1 to enter the nucleus and to specifically counteract the apoptotic action of p53.^10^ Therefore, HIPK2 not only activates the p53-apoptotic pathway by direct phosphorylation but it also reinforces this pathway by phosphorylating Che-1 and inducing its degradation.

It has been demonstrated that HIPK2 is involved in TGF*β*-triggered apoptosis in p53-deficient cells.^36^ As the TGF*β* activation strongly reduces Che-1 cellular levels,^3^ it is possible to speculate that Che-1 levels can be regulated by HIPK2 even in this pathway. Additional experiments will be required to understand this particular aspect.

Finally, it has to be underscored how Che-1 degradation by HDM2 is a part of an important regulatory circuitry between HDM2 and HIPK2/p53 axis. Indeed, upon sublethal DNA damage, HDM2 is involved in HIPK2 degradation,^23^ and in such a way this pathway can contribute to Che-1 stabilization and induction of growth arrest, rather than apoptosis. Conversely, several apoptotic levels of DNA damage lead to HIPK2 activation and Che-1 ubiquitylation by HDM2.

## Materials and Methods

### Cell culture, transfections and analysis

Human HCT116, HEK293, H1299 and U2OS cells were grown in Dulbecco's modified Eagle's medium (DMEM), high glucose, with 10% fetal bovine serum (FBS). Transfections were carried out by BES-calcium phosphate precipitation, as previously described,^1^ or by Lipofectamine 2000 (Life Technologies, Carlsbad, CA, USA) according to the manufacturer's instructions. Transfection efficiency ranged from 30 to 50%. Colony-forming efficiency assay was performed as previously described.^1^ For drug treatment, subconfluent cells were incubated in the presence of ADR (Sigma, St. Louis, MO, USA), MG132 (Calbiochem, Merck-Millipore, Darmstadt, Germany) and z-VAD (Bachem, Bubendorf, Switzerland) at the indicated concentrations.

### Recombinant plasmids and proteins

The following plasmids were used: Myc-tagged Che-1, its partial deletion mammalian expression vectors and Gst-tagged proteins;^1, 7, 9^ Myc-Che-1^T144A^;^9^ pEGFP-HIPK2 and its derivatives;^18^ and the expression vector containing wt HDM2 (kind gift from Dr. Levine) and FLAG-HIPK2.^37^

### Immunoprecipitations, western blot analysis, immunofluorescence and kinase assay

Cell extracts were prepared as previously described.^7^ Solubilized proteins (25 *μ*g) were resolved on MOPS NuPAGE precast 4–12% gels (Life Technologies) and transferred onto PVDF membranes (Millipore, Darmstadt, Germany). Western blotting was performed using the following rabbit polyclonal antibodies: anti-Che-1,^1^ anti-HIPK2 (kindly provided by M.L. Schmitz) and anti-PARP-1 p85 fragment (Promega, Madison, WI, USA). Mouse monoclonal antibodies used were as follows: anti-Myc 9e10 (Life Technologies), anti-HDM2 (MoAb) 2A10 (Ab-2 Calbiochem) and Ab-1 (Oncogene Research Products, La Jolla, CA, USA), anti-*β*-actin (Sigma), anti-*α*-tubulin (Santa Cruz Biotechnology, Dallas, TX, USA), anti-HA probe (sc-8035 and sc-7392 Santa Cruz Biotechnology), anti-FLAG (Sigma) and anti-GFP (Roche, Basel, Switzerland). Secondary antibodies used were goat anti-mouse and anti-rabbit, conjugated to horseradish peroxidase (Bio-Rad, Hercules, CA, USA). Immunoreactivity was determined using the ECL-chemiluminescence reaction (Amersham, GE Healthcare, Buckingamshire, UK), according to the manufacturer's instructions.

Densitometric analyses of immunoblots were performed using the UVITEC Alliance software version 16.07 (serial number 12-630588, Eppendorf, Hamburg, Germany). Ratios were calculated by the following formula:


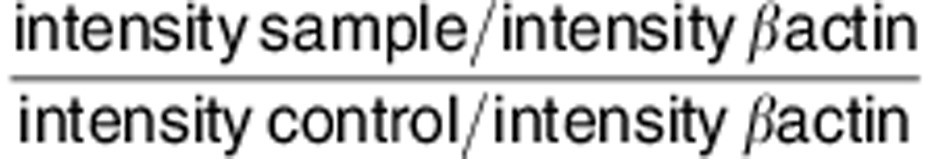


For immunoprecipitation experiments, the cells were lysed by incubation at 4 °C for 30 min in lysis buffer (50 mMTris-HCl, pH 7.4, 250 mM NaCl, 0.1% Triton, 5 mM EDTA and 25 mM NaF). After high-speed centrifugation, lysates were precleared with 20 *μ*l of protein G beads (Santa Cruz Biotechnology) and immunoprecipitated by standard procedures.

Immunofluorescence was performed in human U2OS cells transfected with pEGFP-HIPK2 and pEGFP-K221R. Briefly, cells were fixed in 4% formaldehyde for 15 min and then permeabilized with 0.1% Triton X100 in phosphate-buffered saline (PBS) for 5 min. Anti-Che-1 was used as the primary antibody for immunostaining, and the nuclei were visualized with 4′, 6′-diamidino-2-phenylindole (DAPI). Alexa-Fluor-594-conjugated secondary antibody (Molecular Probes, Life Technologies) was used to reveal the primary antibody.

In kinase assays, immunoprecipitated Flag-HIPK2 was incubated with Gst-fusion proteins for 30 min at 30 °C in kinase buffer (200 mM HEPES pH 7.9, 100 mM MgCl_2_, 1 mM DTT) in the presence of [*γ*^32^P]-ATP. Reactions were stopped by adding 5X sample buffer, and proteins were resolved by SDS-PAGE. Dried gels were analyzed by phosphoimager (Bio-Rad).

### LC-MS/MS analysis

*In vitro* kinase assay was performed by incubating purified Gst-84-163 peptide and Gst-full-length Che-1 recombinant proteins in the presence of HIPK2 active and cold ATP for 1 h at 30 °C in kinase buffer (20 mM HEPES, pH 7.5, 50 mM NaCl, 10 mM MgCl_2_ and 10 mM MnCl_2_). The proteins were resolved by SDS-PAGE and Comassie-stained bands were excised and subjected to in-gel digestion by sequencing-grade modified porcine trypsin (Promega), as previously described.^38^

To identify phosphorylated sites, the peptide mixture was analyzed by liquid chromatography-tandem mass spectrometry (LC-MS/MS) using an Ultimate 3000 HPLC (DIONEX) connected in line with a linear ion trap (LTQ-XL, Thermo Electron). Peptides were separated in a reverse phase, 10-cm capillary column. A data-dependent strategy was used to fragment the five more intense ions present in each full MS scan by collision-induced dissociation. Tandem mass spectra were interpreted through the SEQUEST algorithm,^39^ taking into account the potential for phosphorylation on Ser, Thr or Tyr residues and also manually reviewed. A MS/MS was considered legitimately matched with cross-correlation scores of 1.8, 2.5 and 3, respectively, for one, two and three charged peptides and a probability cutoff for randomized identification of p b 0.001.

### RNA interference

Stable interference by shRNA was performed by transfection of pRetroSuper and pRetroSuper-HIPK2 vectors carrying HIPK2-1376 sequences.^25^ After 24 h from transfection, stable polyclonal populations of Control (Ctr) and HIPK2-depleted cells (HIPK2i-1 carrying HIPK2-1376 sequence) were obtained by selection with 2 *μ*g/ml puromycin. Transient interference by siRNA was obtained by HIPK2i or Che-1i stealth RNAi sequences (a mix of different sequences) and universal negative control stealth RNAi Negative, Medium GC Duplexes (Life Technologies). Cells were transduced by using the RNAiMAX reagent (Life Technologies), as previously described.^30^

## Abbreviations

AATF: apoptosis-antagonizing transcription factor
HDM2: human double minute 2, E3 ubiquitin protein ligase
HIPK2: homeodomain-interacting protein 2
ADR: adriamycin
ΔNp63*α*: amino-deleted p63 isoform
MS: mass spectrometry
z-VAD: caspase inhibitor
MG132: proteasome inhibitor
CtBP: C-terminal binding protein
GST: glutathione S-transferase
Ub: ubiquitine.
